# Clinical and Laboratory Characteristics of Children Referred for Early Puberty: Preponderance in 7-8 Years of Age

**DOI:** 10.4274/Jcrpe.736

**Published:** 2012-12-19

**Authors:** Ayşe Kılıç, Mehmet Sait Durmuş, Emin Ünüvar, İsmail Yıldız, Banu Küçükemre Aydın, Ahmet Uçar, Rüveyde Bundak, Firdevs Baş, Feyza Darendeliler, Fatma Oğuz, Müjgan Sıdal, Ensar Yekeler

**Affiliations:** 1 İstanbul University İstanbul Medical Faculty, Pediatrics, İstanbul, Turkey; 2 İstanbul University Institute of Child Health, Pediatrics, İstanbul, Turkey; 3 İstanbul University İstanbul Medical Faculty, Pediatric Radiology, İstanbul, Turkey

**Keywords:** Pubarche, child, puberty, precox puberty, thelarche

## Abstract

**Objective:** The aim of this study was to evaluate the girls referred to the pediatric outpatient clinic with a presumptive diagnosis of early puberty due to early onset of breast development or pubarche.

**Methods:** Within the study period, we evaluated 289 subjects referred for concerns about early onset of puberty. History, anthropometric data, bone age (BA), hormones including luteinizing hormone (LH), follicle-stimulating hormone (FSH), estradiol, and dehydroepiandrosterone sulfate, as well as pelvic ultrasonography (USG)-derived ovarian and uterine volumes were analyzed.

**Results:** Of the 289 girls referred for early onset of pubertal development, 64 (22.1%) had false alarms for puberty. Of the remaining 225 girls, 41 (18.2%) were diagnosed as premature pubarche, 56 (24.9%) as premature thelarche (PT), and 128 (56.9%) as precocious puberty (PP). Girls with early-onset puberty had more advanced BA, greater uterine and ovarian volumes, and also higher LH values than subjects with PP and PT. Nearly half of these girls were 7-8 years of age. Body mass index (BMI) standard deviation score was significantly higher in the PP cases.

**Conclusions:** There is a need for primary care physicians to be more knowledgeable on puberty and on puberty problems. There seems to be a preponderance of PP in 7-8-year-old children . Increased BMI may have a role in the trend towards earlier onset of puberty

**Conflict of interest:**None declared.

## INTRODUCTION

In girls, precocious puberty (PP) is defined as the onset of breast development under 8 years of age along with advancement of bone age (BA), increased growth velocity, and pelvic ultrasonography (USG) findings consistent with puberty. In most girls, central PP (CPP) is idiopathic and is caused by premature activation of the hypothalamic gonadotropin-releasing hormone (GnRH) pulse generator. Pubic hair can be the first sign of PP. CPP may lead to premature epiphyseal fusion and compromised final height (1,2) in addition to psychological stress (3,4). Thus, early initiation of treatment is of utmost importance (2).

Premature pubarche (PPb) and premature thelarche (PT) are benign, normal variants that can resemble PP but are very slow or non-progressive conditions. PT refers to the isolated appearance of breast development, usually in girls younger than 2 years but may also be seen at 5-6 years of age. PPb refers to appearance of pubic hair without other signs of puberty in girls younger than 8 years.

A thorough history, physical examination, and growth curve review can help distinguish these normal variants from CPP. CPP is gonadotropin-dependent and is characterized by the full spectrum of physical and hormonal changes of puberty as a result of early maturation of the entire hypothalamic-pituitary-gonadal axis. Pseudo PP is much less common and refers to conditions in which the increased production of sex steroids is gonadotropin-independent. CPP often resembles PT that is characterized by isolated early breast development not associated with growth acceleration or bone maturation and thus does not require therapy (5). The incidence of PT is highest in the first year of life, with a second peak after the fifth year (6). The latter increase may represent an "intermediate" entity between isolated PT and CPP (7), also called "thelarche variant", "non-classical PT" or "atypical PT". It is characterized by older age at onset and occasional progression to CPP (8). Physical examination, BA assessment, growth velocity, and the GnRH stimulation test may be helpful in differentiating CPP from PT. It is difficult to distinguish between CPP and PT in their early stages. Another diagnostic challenge arises from the significant increase in obesity prevalence in recent years making it difficult to differentiate between CPP and obesity with or without thelarche. Obese girls who present with pseudothelarche due to increased fat tissue may as well have BA advancement (9,10), while advancement of BA and growth acceleration may be absent in the early stages of CPP. The GnRH stimulation test is considered the gold standard for diagnosis. Yet, despite its high specificity, its sensitivity is low (11,12,13), and the cut-off values of GnRH test are still debatable.

The purpose of the present study was to identify the frequency of referrals of PP cases to a large tertiary pediatric care center. We sought to determine the frequency distribution of subjects with PT, PPb and PP with emphasis on utility of laboratory data.

## METHODS

**Patients:** 289 girls referred to the university clinic because of PP between 01.08.2008 and 31.12.2009 were included in the study. At presentation, 77.9% (n=225) of the study group were found to have on-time alarms for puberty defined as correct identification of the signs of PP before eight years of age by the physician. Of these 225 girls, 56 (24.9%) presented with PT, 41 with PPb (18.2%), and 128 (56.9%) with PP. False alarms for early puberty were observed in the remaining 64 girls (22.1%) who had received a false identification of PP findings by their physician. These cases were either older than 8 years of age or had lipomastia wrongly interpreted as breast tissue.

Medical history, physical examination, antrophometric measurements, pubertal staging, BA, basal levels of sex steroids, follicle-stimulating hormone (FSH), luteinizing hormone (LH) levels, and pelvic USG results were evaluated. Girls referred for evaluation because of the appearance of breast bud and/or pubic or axillary hair were recruited consecutively.

**BA:** Assessment of BA and calculation of the BA standard deviation score (SDS) were performed according to the Greulich-Pyle method (14). Pubertal stage was determined according to Marshall and Tanner (15). Of girls with PT, 67.9% were Tanner stage 2 and 32.1% were Tanner stage 3, while of girls with PP, 31.5% were stage 2 and 68.5% were stage 3.

Height and weight were calculated as SDS for all girls and for both their parents, using the Turkish references (16,17). Body mass index (BMI) was calculated as weight in kilograms/height in m2, and the BMI SDS was calculated according to Turkish references (18).

The diagnosis of PP was based on breast budding before 8 years of age accompanied by one or more of the following findings: menses, pubic hair, accelerated growth velocity, or BA greater than 2 SD above the chronological age. The diagnosis of PT was based on the presence of breast buds in the absence of bone or growth acceleration or pubic or axillary hair. The diagnosis of PPb was based on the presence of pubic or axillary hair in the absence of bone or growth acceleration or breast buds.

Cases that were equivocal on referral were diagnosed after at least 6 months of follow-up by an experienced pediatrician. The diagnosis of PP was based on progression of breast development accompanied by at least one of the following: growth acceleration, BA acceleration, and appearance of pubic hair. All diagnoses were confirmed after at least 1 year of clinical and auxiological follow-up. Girls with chronic disease, bone dysplasia, organic brain disease, congenital adrenal hyperplasia, or other endocrinological abnormalities were excluded.

**Laboratory investigations:** Blood levels of LH and FSH were measured using a solid-phase, two-site chemiluminescent immunometric assay (Immulite 2000, DPC). Serum basal blood levels of estradiol were determined (only in PP and PPb cases) using the double-antibody estradiol procedure (DPC) and of dehydroepiandrosterone sulfate-using chemiluminescent immunometric assay (19).

**Pelvic USG:** Transabdominal pelvic USG scans were performed with a conventional full-bladder technique using 5-MHz real-time sector scanner (Sonoline prima, Siemens) by the same investigator. The following structures were evaluated: (1) Uterus: Length, transverse diameter (width), endometrial thickness, fundal anteroposterior diameter, and cervical anteroposterior diameter. The ratio between the fundal and cervical diameters (FCR) was calculated, and the uterine length was multiplied by the fundal anteroposterior diameter to determine the uterine cross-sectional area (CSA). (2) Ovaries: Height, width, length, number of follicles, and maximum diameter of the largest observed follicle. Ovarian circumference was measured in the transverse position. Uterine and ovarian volumes were calculated according to the formula for ellipsoid bodies: V=longitudinal diameter X anteroposterior diameter X transverse diameter X 0.5233.

The following standard criteria were used to define small for gestational age (SGA): birth weight below the 10th percentile for the gestational age (GA); appropriate for gestational age (AGA): birth weight from 10th percentile through 90th percentile for GA; large for gestational age (LGA): birth weight greater than the 90th percentile for GA (20).

## RESULTS

Of the 128 girls with PP in the study group, 126 (98.4%) were diagnosed as CPP, and 2 (1.6%) - as pseudo PP. All cases of CPP were idiopathic with no abnormality on magnetic resonance imaging (MRI) scans except for one patient with a history of head trauma.

Fifty-seven (44.5%) of the cases with CPP were diagnosed between the ages of 7 and 8 years.

Maternal menarcheal age was 10.6±0.7 years in PP, 12.1±0.9 years in PT, and 13.1±0.4 years in PPb; the difference was statistically significant (p=0.001). Thirty-one (13.8%) of these girls had a history of early menarche in their first-degree relatives.

Mean time of onset of pubertal signs for all cases was age 5.5±1.7 years. Mean BA at presentation was 6.9±2.5 years. When the mean age of onset of pubertal findings was compared in the subgroups, it was seen that PP and PPb cases had an onset at later ages than PT (PT 4.5±1.1 years, PPb 6.2±1.0, PP 5.9±1.0 years). The difference was statistically significant (p=0.001). No difference was found in the mean age of onset of pubertal signs between PPb and PP cases (p=0.18) (Table 1).

BMI of the patients was 15.5±1.6 in PT, 18.2±3 in PPb, and 18.1±2.8 in PP. The difference was statistically significant between PT and PP groups (p<0.001). There was no statistical difference in distribution according to the seasons and the years of presentation (p>0.05). Weight, height, and BMI SDSs were significantly increased in cases with PP than with PT (Table 1). There was no relationship between BMI SDS and hormonal values.

**Birth weight by gestational week:** There were 93 (41.3%) AGA, 109 (48.4%) SGA, and 23 (10.2%) LGA children in the total group. In patients diagnosed as PP, 38 (29.7%) were AGA, 80 (62.5%) were SGA, and 10 (7.8%) were LGA. SGA infants were more common in the PP group.

**BA measurements:** Mean BA was 5.1±2.4 years in patients with PT, 7.0±1.3 years in patients with PPb, and 9.5±1.2 years in the ones with PP. Mean BA in the PP group was found to be significantly higher than in the cases with PPb and PT (p=0.001) (Table 1). The difference between BA and chronological age was also found to be significantly greater in the children with PP as compared to those with PT and PPb, as found in the analysis using the Mann-Whitney U-test and the Wilcoxon method (p=0.001).

Basal hormone levels are shown in Table 2. LH and FSH values in PP cases were found to be significantly higher than in PT and PPb cases (p=0.001).

Ultrasonographic evaluation results are also shown in Table 2. Uterus and ovarian sizes of the cases with the diagnosis of PP were statistically higher than the respective values in the other groups (p=0.001). Five cases of PT and 12 cases of PPb progressed to PP at follow-up.

## DISCUSSION

PP is five times more frequent in girls than in boys. The prevalence of PP is reported to be less than 1% of the normal population (12). During a 2-year period, PP was encountered in 6.2% of patients referred to our general pediatrics department for having a pubertal problem. All of these patients were sent by a doctor or a healthcare institution.

The main results of our study show that there is a high concern about early onset of puberty among primary care physicians, because 22.1% of girls were referred with false alarms for puberty. More education is needed on this topic.

The cases with PP were mainly CPP and most were idiopathic, a finding which is also in compliance with published data (21,22). Interestingly, nearly half of these patients were between 7 and 8 years of age.

Being born SGA and a younger maternal menarcheal age were more closely associated with CPP, a finding which is in compliance with the literature (23,24,25) and can be helpful in differentiating PT from CPP.

BMI SDS was significantly higher in CPP than in PT and PPb cases, indicating that high BMI and obesity may be associated with early onset of breast development (26).

In girls with PP, as expected, BA was more advanced, LH was significantly higher, and uterine and ovarian volumes were also greater than the respective values in girls with PT or PPb. These data are in compliance with published data (27,28,29,30).

In the studies carried out by Della Manna et al (31) and Ilicki et al (32), sex steroids were found to be comparable in cases with PT and PP. In our study, estradiol levels were higher in the PP cases. However, these levels were not found to be statistically different in girls with PP and PT.

Unlike Atay's findings (33), PT children had BMI in normal ranges in out study.

In conclusion, 56.9 % of the patients sent to our general pediatric clinic with complaints of early pubertal onset had PP. Nearly half of the patients were 7-8 years of age. Increased BMI was a prominent finding in girls with PP.

## Figures and Tables

**Table 1:**
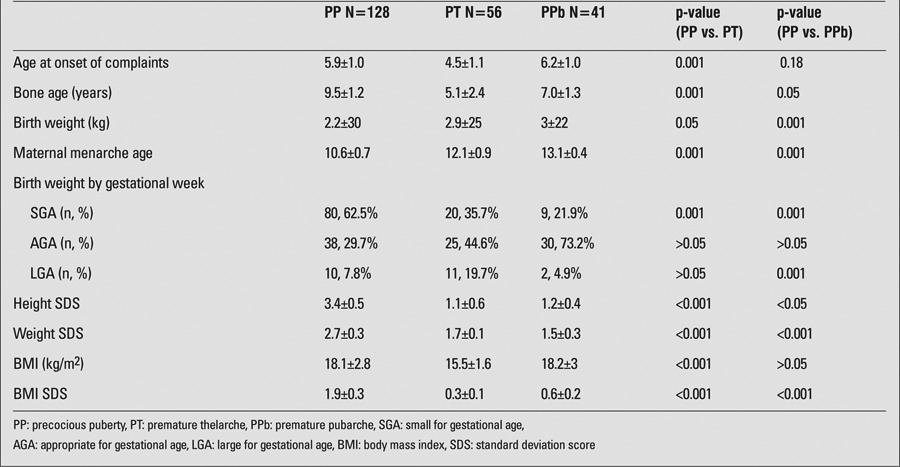
Characteristics of the study group

**Table 2:**
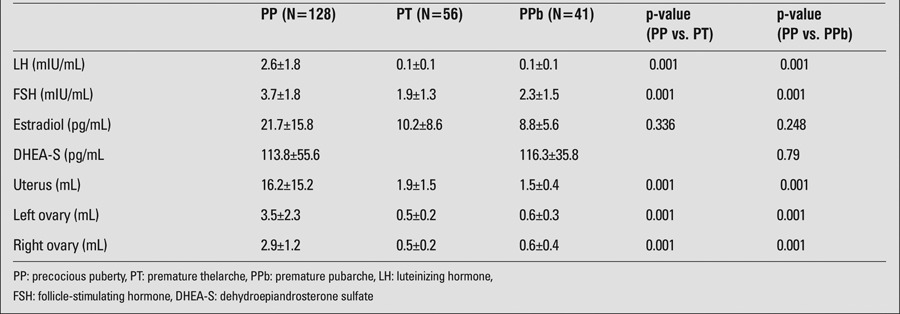
Hormonal levels and ultrasonographic evaluation in PP, PT, and PPb groups
